# Diet and bowel function in children with Hirschsprung’s disease: development and content validation of a patient-reported questionnaire

**DOI:** 10.1186/s40795-023-00737-6

**Published:** 2023-06-28

**Authors:** Lovisa Telborn, Christine Kumlien, Christina Granéli, Irene Axelsson, Pernilla Stenström

**Affiliations:** 1 Department of Clinical Sciences Lund, Lund University, Lasarettsgatan 48, S-221 85 Lund, Sweden; 2 Department of Pediatric Surgery, Skåne University Hospital, Lund, Sweden; 3 Department of Care Science, Malmö University, Lund, Sweden; 4 Department of Cardio-Thoracic and Vascular Surgery, Skåne University Hospital, Malmö, Sweden

**Keywords:** Children, Diet, Gastrointestinal tract, Hirschsprung’s disease, Patient-reported outcome

## Abstract

**Background:**

Although dietary adjustments are recommended frequently for bowel symptoms, evidence of diet’s impact on bowel function is lacking. The aim was to develop a patient-reported outcome instrument, for children with and without Hirschsprung’s disease (HD), to explore experiences of dietary effects on bowel function.

**Methods:**

Children with and without HD and their parents participated. Questionnaire items regarding the impact of diet on bowel function originated from focus group discussions. Specific food items, reported in the literature or in focus groups to cause bowel functional effects, were listed requesting each item’s effect size and effect type. Content validity was tested within two separate semistructured interviews. A pilot test was performed. Assessing comprehension, relevance and wording clarity structurally, revisions were made accordingly. Children’s bowel function was assessed through the validated Rintala Bowel Function Score.

**Results:**

A total of 13 children with and without HD, median age 7 (range 2–15) years, and 18 parents participated in the validation. Each question’s relevance had been ranked highly early in the validation process but most questions needed refining for improving clarity and comprehension. Wordings regarding bowel symptoms and emotions connected to food in particular were perceived to be sensitive and complex. Specifically wording regarding some bowel symptoms (gases, pain) and parental stress emotions (guilt, ambivalence) were, consistent with participants’ opinions, subjected to multiple step revisions. Following the validation process, which included two semistructure interviews with different participants and then a pilot test with a third cohort, a full track overview of changes and rewording made in all steps of the validation process was presented. The final questionnaire then comprised 13 questions assessing foods’ significance for bowel function, emotions, social impact and 90 specific food items’ possible effects and effect sizes on bowel function.

**Conclusions:**

The Diet and Bowel Function questionnaire, enabling answering by children, was developed and the content validated qualitatively. This report presents insights into the whole validation process, declaring reasons for the selected question- and answering options, and their wordings. The Diet and Bowel Function questionnaire can be used as a survey questionnaire to enhance understanding of dietary effects on bowel function in children, and its results can be supportive in improving dietary-treatment programs.

**Supplementary Information:**

The online version contains supplementary material available at 10.1186/s40795-023-00737-6.

## Background

Hirschsprung’s disease (HD) is a congenital intestinal disorder characterized by a lack of ganglion cells in the intestinal wall leading to life-threatening bowel obstruction [1, 2]. HD is treated surgically, often in early childhood, but although the aganglionic bowel is removed, patients with HD frequently report chronic residual morbidity including let-out obstruction, fecal incontinence, pain and flatulence, implying impaired quality of life [1–5]. The underlying cause of bowel dysfunction is multifactorial, e.g. congenital dysmotility, stricture, residual aganglionic segment or anal sphincter damage [1]. Bowel management programs for HD traditionally focus on improving toilet habits and treatment with enemas and/or laxative drugs [1] but, according to the newest HD guidelines, dietary modifications should also be included [2]. This, however, could be a challenge since evidence-based knowledge on which specific dietary treatment to recommend is lacking [6], with very few studies focusing on diet and HD [7, 8]. Diet has been reported to influence bowel function in other pediatric gastrointestinal conditions [9–12] and to be used frequently as a self-treatment in children with constipation [13]. This is despite a lack of evidence for which types of foods actually impact upon healthy children’s bowel function [14]. Self-perceived symptoms related to food groups and food items relevant to food intolerance/allergy (e.g. food items with incompletely absorbed carbohydrates, foods rich in biogenic amines, histamine-releasing foods) have been investigated in a Nordic setting on a population of adult patients with irritable bowel syndrome (IBS) [15], while studies on pediatric populations are absent.

According to our focus group study on dietary effects on bowel function in HD [7], dietary habits play a key role in parental self-treatment of their child’s bowel function, but parents struggled with extensive difficulties in identifying consistent patterns of the effects of specific foods. Based on these results, and to explore children’s experiences further, a patient-reported instrument is required. Such an instrument could be used as a survey questionnaire and bring fruitful insights into the effects of diet on bowel function in children, with and without HD, to explore dietary patterns that could constitute the foundation for interventional studies.

## Methods

### Aim

The aim was to develop and qualitatively test the content validity of a patient-reported outcome (PRO) instrument to be able to explore experiences of dietary effects on bowel function and daily life in children with and without HD.

### Study design and setting

**Fig. 1:**
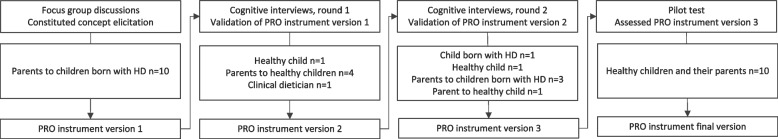
Flow chart of the development and validation process of the Diet and Bowel Function questionnaire. HD, Hirschsprung’s disease

This was a qualitative developmental and content validation of a PRO instrument regarding dietary bowel effects. It followed the recommendations and guidelines in the US Food and Drug Administration (FDA) PRO Guidelines [16] and the International Society for Pharmacoeconomics and Outcomes Research (ISPOR) task force report on PRO instruments for children and adolescents [17]. The development and content validation process included focus group discussions [7], cognitive interviews and a pilot test (Fig. 1).

The study was conducted at a tertiary children’s hospital serving a region with 2 million residents and from 2018 as one out of two national referral centres for HD with a catchment area of 5 million residents.

### Design of the PRO instrument

Items (questions) were generated from the central themes within the focus group discussions [7]. The questions concerned the role of diet in bowel function and daily life in children with HD, as well as specific foods reported to cause effects on the bowel. In addition, foods reported previously to cause bowel effects in other gastrointestinal disorders in Swedish adults [15] (i.e. apricot, cherry, lingonberry, melon, nectarine, peach, apple, avocado, pepper, parsley, celery, chicken, salami, shellfish, wine/beer, fried food, curry, cayenne, chili/tabasco, chamomilla, sesame seeds, hazelnut, peanut, chestnut, almond, brazil nut, walnut, soy) and in children in the USA [9, 10] (i.e. cabbage, sausage, diet soda) were added.

Items for the PRO instrument were created by the main author (L.T.) and representatives from the HD-patient organization. The items were then refined in consensus with the research team, with other patient representatives and with healthy children and their parents. The same wording used by parents within the focus groups was secured in the questionnaire [18]. Response options for all items in the PRO instrument were on a five-grade Likert scale, frequently used in pediatric PRO measures [17], ranging from “Never” to “Always”.

Items aimed to target children and adolescents aged 1–18 years. Parents were instructed to complete the PRO instrument together with their child if the child was younger than 15 years. Adolescents aged 15–18 years old were recommended to try the best they could to answer the questionnaire themselves or otherwise seek help from a parent.

In addition to the items on dietary effects on bowel function and daily life, the PRO instrument also covered patients’ background data and bowel function. Background information included items about other diseases, medications and allergies [6]. Bowel function regarding fecal continence or soiling, constipation, bowel symptoms and social impact, was assessed by the validated Rintala Bowel Function Score (BFS) [19]. Additional items about HD-specific gas and obstruction symptoms were the same as used in previous studies [6, 20]. To be able to generalize the use of the questionnaire, a native English-speaking representative for the HD-patient organization translated the questionnaire and the translation was edited by a professional language reviewer.

### Two rounds of cognitive interviews and a pilot test

Cognitive interviews were carried out to assess the content validity which was the respondents’ perceptions of each item’s relevance, clarity and comprehension [16, 17, 21]. The results from the cognitive interviews were used for refinements of the instrument. Securing a diversity of participating responders in the cognitive interviews, participants were purposively selected regarding age, gender and whether they had HD or not. To confirm patient understandability of an item, inclusion to the cognitive interviews followed the recommendations of interviewing 7–10 participants, planning for further inclusion until saturation is obtained [22]. The patient representatives were recruited through the HD patient organization and healthy children through randomly selected pre-schools and primary schools. Healthy was defined as being without any gastrointestinal or urological malformations, any known gastrointestinal, nephrological, metabolic or other diseases, or having medically treated allergies. Children and parents were asked to read and fill in the questionnaire before the interview. The interviews were held individually by the main author (L.T) at a place decided upon together with the participant. The interviews were performed in a semistructured manner following the interview guide presented in Table 1, including 1: Comprehension and relevance of each item; 2: Wording; and 3: Overall assessment.

**Table 1:** Cognitive semi-structured interview questions

**Comprehension and relevance**	
1	In your own words, can you explain what the question means to you? (open answer)
2	Do you find the question easy or hard to understand? (Easy/Hard)
3	Do you find the question easy or hard to answer? (Easy/Hard)
4	Do you find the question relevant to ask? (Yes/No)
5	Do you have any comments on the question?
6	Read each of the options for answers, and tell me what they mean to you?
7	What do you think about the options for answers?
8	Could you find your first-choice answer among the options for answers?
9	Were there any words that you found hard to understand?
**Wording**	
1	What do you think about the form of questioning “you/your child”?
2	What does the term “bowel function” mean to you? Would you prefer another term? If yes, which term?
3	What does the term “psychological” (round 1)/”emotional” (round 2) mean to you? Would you prefer another term? If yes, which term?
**Ovarall assessment**	
1	What do you think about the instructions to fill in the questionnaire?
2	How long did it take for you to fill in the questionnaire?
3	Do you have any other comments that could help us improve the instrument?

Collecting high-quality comprehension of the respondent’s interpretation of each question, the interviewer used a verbal probing approach [21]. Participants’ answers were recorded by field notes. Interviews were conducted in two rounds, with different participants in each round. After each round, the questionnaire underwent refinements in consensus within the research team and with patient organization representatives.

After two rounds of cognitive interviews, the pilot version of the PRO instrument was tested by 10 healthy children [23]. Children in the waiting area at the Day Surgery Unit were invited to participate. The PRO instrument was handed out with oral and written instructions, with the offer of asking the researcher about anything that was unclear.

### Data analysis

The focus groups were analyzed by content analysis [7]. The cognitive interviews were analyzed by informal analysis [22]. Item-oriented results from the cognitive interviews guided decisions about keeping, modifying or deleting items. Questions for which respondents identified difficulties with comprehension, clarity or relevance were revised based upon participants’ responses and suggestions. Quantitative data were reported as total numbers (n) and medians (range) for continuous variables and as total numbers for discrete variables.

### Ethics

All methods were carried out in accordance with relevant guidelines and regulations. The study was approved by the Swedish Ethical Review Authority (registration number: 2018/720). Participants received age-adapted oral and written information. Informed consent was obtained from all subjects and/or their legal guardians.

## Results

**Fig. 2:**
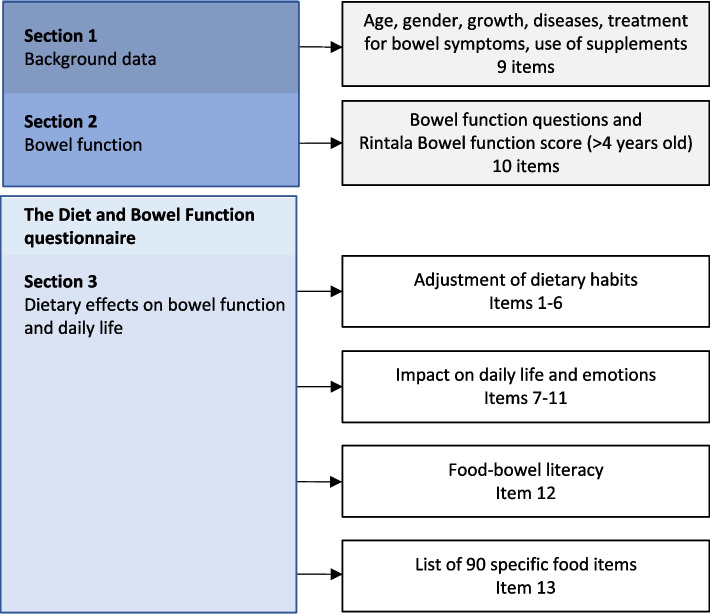
Design of the instrument decided in collaboration with patient representatives and healthy participants. Questions and answers in the Diet and Bowel Function questionnaire (Section 3) were validated within this study

The PRO instrument in its whole included background data, assessment of bowel function and then the questionnaire objected to the qualitative development and validation here presented (Fig. 2). The results of the content validation process are displayed in a step-by-step overview in Additional file 1. Details of each step are presented below in the cognitive interviews and of the pilot study.

The validation process and the following revisions led to the final version of the Diet and Bowel Function questionnaire, presented below in Table 2. Questions and answers regarding background and bowel function which were also asked about, but not validated, are displayed in Additional file 2.

**Table 2:** Items and answering options included in the Diet and Bowel Function questionnaire

**Dietary effects on bowel function and daily life**	
1	Would you agree that your diet affects your stomach? (e.g. constipation, diarrhea or bloatedness) (No, never/Yes, sometimes/Yes, often/Yes, always/Not currently but I have in the past/Please, explain how)
2	Would you agree that how you eat affects your stomach? (No, never/Yes, sometimes/Yes, often/Yes, always/Not currently but I have in the past/Please, explain how)
3a	Do you adjust your diet for your stomach’s sake? (No, never/Yes, sometimes/Yes, often/Yes, always/Not currently but I have in the past/Please explain how)
3b	If yes: Why? (Laxative effect/Constipating effect/Less gases/Other. If so, what?)
4	Do you choose specific types of food to help your stomach? (Yes/No)
5	Do you avoid specific types of food to help your stomach? (Yes/No)
6	Is there anyone else in your family that adjusts their diets to help their stomach? (Yes/No)
7	Does your diet limit you (in school, when you are with friends or in general)? (I’ve never thought about it, so I don’t find it relevant/No, never/Yes, sometimes/Yes, often/Yes, always/Not currently but I have in the past/Please explain how)
8	Do you think about how your diet affect your stomach? (No, never/Yes, sometimes/Yes, often/Yes, always/Not currently but I have in the past/Please explain how)
9	To parents: Do you think about your child's diet and how it affects his/her stomach? (No, never/Yes, sometimes/Yes, often/Yes, always/Not currently but I have in the past/Please explain how)
10	Does your diet affect you emotionally? (No, never/Yes, sometimes/Yes, often/Yes, always/Not currently but I have in the past/Please explain how)
11	To parents: Does your child’s diet affect you emotionally? (No, never/Yes, sometimes/Yes, often/Yes, always/Not currently but I have in the past/Please explain how)
12a	Would you be interested in finding out more information about how your diet affects your stomach? (Yes/No)
12b	If yes: Where or who would you turn to to find out more information? (Open answer)
**The effect of specific food items on bowel function**	
13a	Does the food item affect your stomach? (For every food item: Yes/No/I don’t know)
13b	If yes: In which way? (For every food item: Laxative effect/Constipating effect/Gives gases/Gives pain/Other. If so, what?)
	Food items listed: Fruits: pineapple, orange, apricot, banana, clementine, strawberry, kiwi, cherry, lingonberry, melon, nectarine, peach, plum, pear, dried fruit, grapes, apple Vegetables: avocado, cauliflower, broccoli, beans, cabbage, lentils, onion, corn, carrot, pepper, parsley, potato, rhubarb, celery, fruit peel, asparagus, mushroom, tomato, peas Dairy: cream, ice cream, lactose-free milk, milk, cheese, butter, yoghurt Bread, flour, rice: bread with grains/seeds, Swedish cracker, cornmeal, pasta, rice, flour, white bread Meat, fish, egg: fish, pork, beef, sausage, chicken, salami, shellfish, egg Sweets and snacks: pastry, chips, chocolate, candy, popcorn, rice cakes, pretzel sticks Beverages: soda (with sugar), soda (free from sugar), water, carbonated drink, wine/beer, formula Cooking effects: deep fried food, spicy food, fried food, soup Spices and seeds: curry, cayenne, chili/tabasco, chamomile, sesame, sunflower seeds, poppy seeds Nuts etc.: cashew, hazelnut, peanut, chestnut, almond, brazil nut, walnut, soya Other food item (Open answer)

### Version 1: Cognitive interviews

The overall theme deriving from the focus group discussions [7] was “*Diet is a strong influencer on bowel function in HD*” and comprised the following three categories. The first category *“Striving to regulate bowel function through dietary strategies”* derived from participants’ experiences of how, and to what extent, diet impacts upon bowel function in children with HD, and how participants used different food items to regulate bowel function. This category resulted in items 1–6 and item 13 concerning bowel effects by specifically mentioned food items (Fig. 2). Frequently mentioned food items to influence bowel function included within item 13 were fruits, vegetables, dairy products, dietary fiber, white bread, rice, pasta, fats, sweets and popcorn. Specific bowel symptoms, caused by food, that were mentioned in focus group discussions were gas production, pain, constipating and laxative effects, used as alternative answers in items 3 and 13.

The second category deriving from focus groups was *“Restricting diet to control bowel function impact on family- and daily life”* including if, and to what extent, food selection and food adjustments impacted upon participants’ daily life and emotional status. Using exact wording used by parents, the second category encompassed items 7–11 (Fig. 2). The third category “*Wishing for dietary and nutritional guidelines for facilitating self-treatment*” concerned food-associated bowel function literacy and formed item 12 (Fig. 2).

The first version of the PRO instrument was then validated by individual cognitive interviews with six participants: one clinical dietician, one 12-year-old healthy boy, and four parents (two mothers and two fathers) to healthy children: two boys and two girls, median age 7.5 (range 3–12) years. Interviews lasted 45–60 min each. According to the interview structure (comprehension and relevance, wording and overall assessment), participants reported that items, in general, were easy to understand and were of high relevance. Regarding answer options, the distinguishing between the options in the 5-point Likert scale was reported to be difficult, exampled by one participant who said: *“It’s difficult to tell the difference between ‘Rarely’ and ‘Sometimes’”*. As a result, the scale was converted to a 4-point Likert scale. Participants also requested to, in addition to the given alternatives, have the choice of answering by using their own words. Therefore, the option “*Please explain how*” was added to 10 items.

Refinements of wording were required for all items, especially for children’s understanding of bowel function wording. The 12-year-old boy said: “*Bowel function. I don’t know what that means, I have never heard that word before*”. Considerable effort was made to comprehend crucial words such as “bowel function” which was changed to “stomach*”,* while “diet and meal time habits”/ “different/specific types of food or drink”/ “food routines” were simplified to the terms “diet” and “meal-time habits”. Portion sizes or regular/irregular meal times were word-revised to “how you eat”. Initial complicated wording in the item “If you/your child could avoid all types of food and drink that cause you/your child problems, how often do you think you would suffer from stomach problems, such as pain or bloatedness or bowel function problems such as constipation, etc.?” was revised to: “Do you think it would be possible to avoid stomach or bowel problems by adjusting your diet?” (see Additional file 1, item 6 in PRO instrument version 1). Also, wording of feelings related to food was reported to be complicated and required extensive discussions. Therefore item 10: “Does your/your child’s stomach or bowel problems affect you/your child psychologically?” was revised to: “Does your diet affect you emotionally?” (Additional file 1).

Assessing the PRO instrument in its entirety, participants requested more detailed instruction on how to fill in the questionnaire, and on what proportion of participation that parents and children, respectively, should take in answering, when answering together.

### Version 2: Cognitive interviews

The second version of the PRO instrument was assessed by a second round of individual cognitive interviews with another six respondents: one 15-year-old boy with HD, one 15-year-old healthy boy, three parents (two fathers and one mother) to children with HD who were three boys with a median age of 5.3 (range 3–8) years, and one parent to a healthy 6-year-old girl. Interviews lasted 30–45 min each. Assessing comprehension and relevance, the questions were reported to be both easy to understand and to answer, and of high relevance for examining the role of diet on bowel function and daily life. Wording revisions were required for 6/12 questions. In accordance with respondents’ requests, the question: “How often do you…?” was replaced by: “Do you…?”, and the answering option was changed from: “Never” to “No, never” (questions 3, 7 and 8). As a result of the difficulties in finding generally accepted wordings for emotions, the question: “Does your diet situation affect you emotionally?” once again was discussed thoroughly by all participants. Retaining the word “emotional” it was reworded to: “Does your diet affect you emotionally?” (questions 10 and 11). One question was changed to be an active voice sentence: “Would you agree that your stomach is affected by different types of food?”; was changed to: “Would you agree that your diet affects your stomach? (e.g. constipation, diarrhea or bloatedness)”. For bowel effects: “Facilitate to poop” was replaced by: “Laxative effect” and “Constipating effect” (item 3).

The question: “Do you think it would be possible to avoid stomach or bowel problems by adjusting your diet?” was finally omitted as a result of the fact that one participant perceived it to be offensive, signaling a lack of good parenting: *“The question put pressure on you as a parent. If you haven’t experienced or heard about Hirschsprung’s disease, you would think it was a diet-related disease”.* Instead one question about family dietary habits was added because the dietary adaptations were requested to be set within a family concept: “Is there anyone else in your family who adjusts their diet to help their stomach?” (item 6).

In the overall assessment, several participants suggested that since food was mostly selected and cooked by parents, the PRO instrument should be answered by both the child and parent, no matter what the age of the child (younger than 18 years old). The instructions were changed accordingly.

### Version 3: Pilot test

In the pilot test, 10 healthy children (five girls and five boys) with a median age of 7 years (range 2–15 years) and their parents participated. To emphasize the importance of answering question 13, since one respondent misinterpreted instructions and therefore did not answer that question, an additional sheet was placed between questions 12 and 13. To clarify between: “Do you choose specific types of food to help your stomach?” and: “Do you avoid specific types of food to help your stomach?” these two questions were changed from paired in question 13 to separate single questions (questions 4 and 5). (Additional file 1). The question about dietary adjustments (question 3) was re-written from a primary question: “If you adjust diet for your stomach’s sake, what are the reasons?” to: “Do you…” with a dichotomous answer option yes/no, with a follow-up question: If yes: why? After these revisions, the research group decided on the final version of the PRO instrument (Table 2). The Diet and Bowel Function questionnaire including background data and bowel function score is to be found in an additional file (see Additional file 2).

### The final version

The above-described validation precess and revisions of questions 1–13 regarding dietary effects on bowel function and daily life led to the final version of the Diet and Bowel Function questionnaire, Table 2.

## Discussion

Within an unexplored area, as with the effect of diet on bowel function, validated PRO instruments are imperative for increasing insights. Using well-established qualitative methods, the Diet and Bowel Function questionnaire was hereby developed and its content was validated. By including children, their parents and the HD patient-organization throughout the whole process, the methods used ascertained patient involvement and clinical relevance. The central themes of the questionnaire, and wording, were rooted thoroughly in parental focus group discussions. Then, within repeated semistructured cognitive interviews and a pilot, the questionnaire’s items passed through multiple revisions, refinings and rewording, here described.

According to good research practice, the development of PRO instruments should involve representants from the intended population already from the stage of understanding the relevance of the theme to be studied – the concept elicitation [16, 17]. In line with this, the focus group discussions, as used for this study, are a recommended and a commonly used way of generating PRO instrument items [16, 17, 24, 25]. Focus groups are also reported to provide the opportunity to capture patients’ specific language in detail when describing their situation [17]. We confirm that the multiple focus groups efficiently encircled HD-specific relevant areas and themes, and largely aided in the item phrasing. Furthermore, the focus groups did not only provide relevant information on specific food items that affected children’s bowel function, but also captured emotional and social experiences and perspectives of dietary habits [7].

Cognitive interviewing, as used in this study, is a well-established method to validate new instruments qualitatively and to improve their design [16, 17, 21]. The two cognitive interview rounds used in this content validation secured relevance and increased comprehension through multiple word refinings focusing specifically on a language suitable for children. One proven advantage of cognitive interviews is their efficacy for sustainability also in quantitative field situations [22]. Buers et al.showed that when comparing outcomes of qualitative cognitive interviews with the question response in quantitative field tests, cognitive interviewing was found to be three times more sensitive in identifying problematic questions [26]. The number of cognitive interviews has been reported to be determined by time and resources, but still most critical problems have been reported to be detected through a small number of cognitive interviews [27] and samples of 5–15 participants per round are used commonly [22]. In addition, it has been shown that small numbers of cognitive interviews seem to expose proportionally more high-impact problems, and the number of unique problems revealed is most striking in small sample sizes [28].

The informal analysis of the cognitive interviews used in our content validation was performed according to the original method described [22]. In contrast to formal analyzes, in which data reduction is reported to be a risk due to the coding method, informal analyzes are reported to generate more detailed, complex and sensitive information [22, 26]. Specifically in this study, the informal analyzes allowed detection of detailed information about the participants’ comprehension and interpretation of specific items.

In the content validation process, it became apparent that some questions, especially the complex ones about emotions, needed to be discussed back and forth, and some were refined several times before attaining good comprehensibility. This is in congruance with the fact that cognitive interviews are known to identify very complex topics [22]. This was illustrated by the most complex question in our questionnaire: “*Does your diet affect you emotionally*?” which, especially among participants without gastrointestinal symptoms and dietary restrictions, gave rise to complex discussions on wording and comprehension. The question derived from focus groups’ discussions in which emotions connected to food was a central theme and one of the most pertinent issues among parents of children with HD. Cognitive interviews are also known to identify topics that are so complex that they *cannot* be studied within a PRO instrument. This could be illustrated by the second most complex question: “*Do you think it would be possible to avoid stomach or bowel problems from adjusting your diet?”.* This question derived from the focus groups’ consistent discussion about participants’ strong belief in the potential of diet to affect bowel function and their endeavors to find the perfect diet for their child’s bowel condition [7]. One participant in the cognitive interviews had strong opinions that the question casted doubt on good parenthood, so the question was omitted. To omit a question, although only one single participant had opinions about it, is in accordance with general recommendations that emotionally charged and potentially offensive questions should be given strong consideration of being deleted, as a result of their potential threat to weaken the instrument’s overall credibility [17].

When considering the age of children answering the Diet and Bowel Function questionnaire, the child’s/adolescent’s dependence on their parents, with regard to food selection and cooking, became evident in the cognitive interviews. Since both parents and children/teenagers asked for the opportunity of answering questions together, instructions were changed to allow answering together for children of up to 18 years of age. Such adaptation is in line with the recommendations that each PRO instrument’s specific age cut-offs should be set separately [17].

There is a gap in knowledge regarding whether dietary GI effects differ between children with HD and otherwise healthy children experiencing GI symptoms. The cognitive interviews we conducted indicated that children with HD experienced that more food items induced GI effects to a greater extent than healthy children and their parents did. The patients with HD talked in a more lively manner and with greater recognition of the impact of diet on bowel function and daily life, compared to the healthy children. The cognitive interviews also indicated that children with HD have different experiences of how diet impacts upon their bowel function in daily life. This needs to be confirmed quantitatively.

Strengths of the study include a true patient-oriented and multi-professional approach, by involving children and/or their parents and diverse health-care professionals in the research team. Another strength is that the revisions of complex items from the first round of cognitive interviews were tested in a second round of cognitive interviews, thereby confirming a correct revision direction. Content validity was ensured by concept elicitation involving the target group in both focus group discussions and in multiple and comprehensive cognitive interviews. Objectivity was ensured by researcher triangulation.

Limitations of the study were that all participants in the cognitive interviews were of Nordic origin, meaning that the instrument might be of some limited use within other cultures with other dietary habits. In order to extend the use of the instrument to other societies with different food cultures, it might be beneficial to revise the list of food items so that they are less comprehensive and more general. This calls for further confirmatory work. Regardless of the high number of food items included in the questionnaire, there are food items (e.g. fermentable oligosaccharides, disaccharides, monosaccharides and polyols [FODMAPs]), food components (e.g. sugar alcohols), eating behavior (e.g. chewing gum), and diets used in bowel management (e.g. low-fiber diet) that were not specified in the questionnaire. Although respondents were encouraged to answer from their personal experiences in the free text, the lack of specific examples may constitute an answer bias. Furthermore, the question regarding special diets, citing only vegan or vegetarian diets, and not other diets, might imply a response bias. These limitations need careful consideration when using the questionnaire and interpreting its results. Another limitation was that we did not study the reliability of the instrument over time or in a larger field. Cognitive interviews are often used in combination with classical psychometric tests and in field testing [26, 29–31]. Classical psychometric test theory includes the ability to detect change over time [17, 29–31] but this requires basic knowledge and evidence within the area to be explored. Since basic knowledge, and especially evidence, are lacking in the effects of diet on the bowel in children with and without HD, assessing differences in groups of children with HD, e.g. young/old, mild HD/severe HD (group-validity), or possible change in bowel effects with increasing age (ability to detect change), was not applicable in the development of this instrument. Further multicenter studies with a larger sample size and classical psychometric testing could be important to clairify findings and increase the validity.

## Conclusions

The Diet and Bowel Function questionnaire enabling involvement of children is hereby developed and its content validated qualitatively. It is, according to responders, comprehensive and answered easily. This PRO instrument can be used as a survey questionnaire to enhance our understanding of dietary habits’ potential impact on bowel function in children, with the aim of improving evidence-based support to patients’ self-treatment. The instrument might also be useful as a clinical tool for equal assessment of issues that are often referred to by patients with HD.

## Supplementary Information

**Additional file 1.**

**Additional file 2.**

## Data Availability

The datasets generated and analyzed during the current study are not publicly available due to maintenance of confidentiality and protection of each participant's personally identifiable information, but are available from the corresponding author on reasonable request.

## Abbreviations

HD: Hirschsprung’s disease
PRO: Patient-reported outcome
IBS: Irritable bowel syndrome
