# Supplementary material for: Diet and bowel function in children with Hirschsprung’s disease: development and content validation of a patient-reported questionnaire
Source: BMC Nutr. 2023 Jun 28;9:78. doi: 10.1186/s40795-023-00737-6 (PMC10308757; doi:10.1186/s40795-023-00737-6)
Supplement: Supplementary file 1 — Additional file 1. [file 40795_2023_737_MOESM1_ESM.pdf]

Additional file 1. Overview of revisions of PRO instrument items about diet and bowel function, based on cognitive interviews and field testing

Items in PRO instrument Version 1 derived from Focus groups	Cognitive interview findings Round 1	Items in PRO instrument Version 2	Cognitive interview findings Round 2	Items in PRO instrument Version 3	Field test findings	Items in PRO instrument Final version
**Would you agree that you/your child's bowel function (e.g. constipation, diarrhea or bloatedness) is affected by different types of food?**<br>(Item 1)	**Comprehension and relevance** Participants found the item relevant and easy to understand.<br>**Wording** "Bowel function" gave rise to multiple interpretations and was hard to understand, especially for children. "*I don't know what that means, I have never heard that word before*". "*For me bowel function means the stomach and the stool/feces. The word 'stomach' is better to use.*"<br>**Change:** "Stomach" replaced "bowel function".	**Would you agree that your stomach (e.g. constipation, diarrhea or bloatedness) is affected by different types of food?**<br>(Item 1)	**Comprehension and relevance:** Participants found the item highly relevant and easy to understand and answer.<br>**Change:** The item was changed to be an active voice sentence. Explanations in the parentheses were placed in the end of the item, as suggested by participants.	**Would you agree that your diet affects your stomach? (e.g. constipation, diarrhea or bloatedness)**<br>(Item 1)	No problems reported.	**Item 1**<br>**Would you agree that your diet affects your stomach? (e.g. constipation, diarrhea or bloatedness)**
**Would you agree that meal-time habits (factors such as portion sizes or regular/irregular mealtimes) affect you/your child's bowel function?**<br>(Item 2)	**Comprehension and relevance** The item was considered easy to understand and was deemed highly relevant.<br>**Wording** "Meal-time habits" was hard to understand for the child, and gave rise to discussions about wording among the adult participants. "*Meal-time habits is a difficult word. Change to how you eat*"<br>**Change:** "Stomach" and "how you eat" replaced "bowel function" and "meal-time habits".	**Would you agree that how you eat affects your stomach?**<br>(Item 2)	**Comprehension and relevance** Participants described the item as easy to understand. Some participants who didn't experience any bowel problems found it hard to answer and considered it to be less relevant. "*We haven't seen a difference for our daughter but for other parents the question may be relevant*".<br>**Change:** Addition of answer alternative "Not relevant", as suggested by participants.	**Would you agree that how you eat affects your stomach?**<br>(Item 2)	No problems reported	**Item 2**<br>**Would you agree that how you eat affects your stomach?**

**How often do you think about how your diet and your mealtime habits affect you/your child's bowel function?**<br>(Item 3)	**Comprehension and relevance:** Several participants with healthy children found the item hard to answer due to the fact that they had never thought about the issue. Therefore, the relevance was considered moderate, since the item was thought to be less relevant for healthy children.<br>**Wording:** The wording was perceived as complicated by children. A suggestion was made to change the wording to "*How often do you think about how your diet affects your stomach*?" Several participants suggested that the item should be put in another place in the PRO instrument.<br>**Change:** "Stomach" replaced "bowel function". "Daily food/meal routines" was omitted. Order changed from 3 to 7. The item was divided and directed to children and parents, respectively.	**How often do you think about how your diet affects your stomach?**<br>(Item 7)	**Comprehension and relevance:** All participants found the item easy to understand and answer and considered the item to be highly relevant. One participant, a parent to a child born with HD, expressed "*The question is to do with quality of life. Our son knows what he can and can't eat but doesn't seem to be bothered or overthink about it*".<br>**Change:** The item was reworded due to rewording of response options.	**Do you think about how your diet affects your stomach?**<br>(Item 6)	No problems reported	**Item 8**<br>**Do you think about how your diet affect your stomach?**
		**To parents: How often do you think about your child's diet and how it affects his/her stomach?**<br>(Item 8)	**Comprehension and relevance:** All participants expressed the relevance of asking the child and the parent by using separate items. Some participants explained that they would have given different answers to their child. The teenage participants thought that they would skip the item "To parents", when answering the questionnaire.<br>**Change:** The item was reworded due to rewording of response options.	**To parents: Do you think about your child's diet and how it affects his/her stomach?**<br>(Item 7)	No problems reported	**Item 9**<br>**To parents: Do you think about your child's diet and how it affects his/her stomach?**

**How often do you choose/avoid specific types of food or drink to avoid stomach or bowel problems?** (Item 4)	**Comprehension and relevance:** The item was interpreted as intended and was found to be easy to understand. **Wording:** Complicated wording. **Change:** According to suggestions from participants: "*How often do you adjust your diet for your stomach's sake*?"	**How often do you adjust your diet for your stomach's sake?** (Item 3)	**Comprehension and relevance:** All participants found the item relevant and easy to understand and answer. A participant, born with HD, expressed "*This is a good question, it leaves room to reflect*". **Change:** The item was reworded due to rewording of response options.	**Do you adjust your diet for your stomach's sake?** (Item 3)	No problems reported	**Item 3a** **Do you adjust your diet for your stomach's sake?**
**If you choose/avoid specific types of food or drink, what are the reasons?** (Item 5)	**Comprehension and relevance:** The item was interpreted as intended and was found to be easy to understand by participants. **Wording:** Complicated wording. **Change:** According to suggestion from participants: "*Change 'choose/avoid' to 'adjust'* "	**If you adjust your diet for your stomach's sake, what are the reasons?** (Item 4)	**Comprehension and relevance:** In comparison to the former items, participants explained that this item was more informative, giving rise to self-reflection, and therefore was highly relevant.	**If you adjust your diet for your stomach's sake, what are the reasons?** (Item 4)	No problems reported **Change:** After discussion within the research team, the item was changed to be a follow-up question, to make it clearer.	**Item 3b** **If yes: Why?**
						**Item 4** **Do you choose specific types of food to help your stomach?**
						**Item 5** **Do you avoid specific types of food to help your stomach?**
**If you/your child could avoid all types of food and drink that cause you/your child problems, how often do you think you would suffer with stomach problems such as pain or bloatedness or bowel function problems such as constipation, etc?** (Item 6)	**Comprehension and relevance:** All participants interpreted the item as intended, as expressed by one participant: "*Do you think that all of your problems could be resolved if you have the right diet*?". The item was found to be relevant, and reflections were made on the impact of food adjustments, as expressed by one participant: "*Observe and take note if they think that diet is what affects their stomach problems*". **Wording:** Complicated wording. **Change:** According to suggestions from participants: "*Do you think it would be possible to avoid stomach or bowel problems from adjusting your diet?"*	**Do you think it would be possible to avoid stomach or bowel problems from adjusting your diet?** (Item 5)	**Comprehension and relevance:** The participants explained that the item was easy to understand. Several participants reflected on the item as important, but one participant declared that the item was potentially putting pressure on her as a parent "*If you haven't experienced or heard about Hirschsprung's disease you would think it was a diet-related disease*". **Wording** It was described as hard to answer due to its hypothetical wording. **Change:** The cited reflection led to the decision to omit the item.			

**Does your/your child's diet and food routines limit you/your child in school, when you/your child are with friends or in general?**<br>(Item 7)	**Comprehension and relevance:** Participants expressed difficulties with comprehension, related to the wording.<br>**Wording:** Complicated wording<br>**Change:** According to a suggestion from participants: *"Does your diet limit you (in school, when you are with friends or in general)?"*	**Does your diet limit you (in school, when you are with friends or in general)?**<br>(Item 6)	**Comprehension and relevance:** Participants declared the item to be relevant, easy to understand and answer. Participants, especially parents to children with HD found the item highly relevant "*As a mum to a child with Hirschsprung's disease you find it harder and consider a lot of times before leaving your child with someone else*". Another participant reflected on the question "*More than likely most relevant for those who have problems, but it is good that there are alternative answers for those who don't suffer from the problems*".	**Does your diet limit you (in school, when you are with friends or in general)?**<br>(Item 5)	No problems reported	**Item 7**<br>**Does your diet limit you (in school, when you are with friends or in general)?**
**Does your/your child's stomach or bowel problems affect you/your child psychologically?**<br>(Item 8)	**Comprehension and relevance:** Participants described the meaning of the item as intended. Still, problems with clarity appeared, since the item assumes that the respondent has bowel dysfunction.<br>**Wording** A new wording was introduced: "Stomach or bowel problems", which was suggested to be consistent in wording for the entire PRO instrument. The participants were asked to describe the meaning of "psychologically", giving rise to extensive discussions on wording and comprehension.<br>**Change:** Since the participants had great difficulties in finding a suitable description of the meaning, and the answers were not consistent, the item was reworded, according to a suggestion from a participant: "*Does your diet situation affect you emotionally*?".	**Does your diet situation affect you emotionally?**<br>(Item 9)	**Comprehension and relevance:** Most participants found the item understandable. One participant expressed: "*If you do suffer with problems and you think about it all the time, it must affect your quality of life*". The item was thought to be hard for a child to answer, and it was thought to be more relevant for children with bowel symptoms.<br>**Wording:** Although the item was reworded to Questionnaire version 2, the word "emotional" appeared complicated. Participants reflected on the possibility of "emotional" to include both positive and negative spectra of feelings. Participants tried to find another wording but found it hard. Overall, "emotional" was thought to best describe the intention.<br>**Change:** It was suggested that "Diet situation" be reworded to "diet".	**Does your diet affect you emotionally?**<br>(Item 8)	No problems reported	**Item 10**<br>**Does your diet affect you emotionally?**

**To parents: Does your child's stomach or bowel problems affect you psychologically?**<br>(Item 9)	**Relevance:** All participants expressed the relevance of asking both the child and the parent.	**To parents: Does your child's diet situation affect you emotionally?**<br>(Item 10)	**Comprehension and relevance:** Participants found the item relevant and easy to understand and answer. "*Through the question you can show that it's ok to be affected, it's good*".	**To parents: Does your child's diet affect you emotionally?**<br>(Item 9)	No problems reported	**Item 11**<br>**To parents: Does your child's diet affect you emotionally?**
**Do you wish that there was more information available about how your/your child's diet affects your/your child's bowel function?**<br>(Item 10)	**Wording:** Complicated wording<br>**Change:** According to a suggestion from a participant: "*Would you be interested in finding out more information about how your diet affects your stomach?"*	**Would you be interested in finding out more information about how your diet affects your stomach?**<br>(Item 11a)	**Comprehension and relevance:** Participants found the item relevant and easy to understand and answer. "*The question is crystal clear, what it means*". Participants asked if the research team planned to give further information to study participants answering "yes" to the item.	**Would you be interested in finding out more information about how your diet affects your stomach?**<br>(Item 10a)	No problems reported	**Item 12a**<br>**Would you be interested in finding out more information about how your diet affects your stomach?**
**If you were looking for more information about how your diet affects your/your child's bowel problems where would you look or who would you turn to for help?**<br>(Item 11)	**Comprehension and relevance:** Participants expressed that the question repeated the former question.<br>**Change:** According to a suggestion from a participant, the item was changed to be a follow-up question to the former question.	**If yes: Where or who would you turn to to find out more information?**<br>(Item 11b)	See above on question 11.	**If yes: Where or who would you turn to to find out more information?**<br>(Item 10b)	No problems reported.	**Item 12b**<br>**If yes: Where or who would you turn to to find out more information?**
				**Is there anyone else in your family that adjusts their diet to help their stomach?**<br>(Item 11)	No problems reported.	**Item 6**<br>**Is there anyone else in your family that adjusts their diet to help their stomach?**
**Does the food item affect your/your child's stomach?**<br>(Item 12a)	**Comprehension and relevance:** Participants explained the table with food items to be complicated at first sight. There was a high number of food items (90), but the participants explained that once they understood the structure, the question was easy to answer. Participants suggested that the item might be time consuming, since the respondent might start to reflect upon each of the food items. Participants considered the item to be highly relevant.<br>**Change:** The numerous answering	**Does the food item affect your stomach?**<br>(Item 12a)	**Comprehension and relevance:** Participants explained that all items were relevant and easy to understand. At first sight, some participants explained, the table gave the impression of being complicated, but they found it easy to answer once they began.	**Does the food item affect your stomach?**<br>(Item 12a)	**Comprehension and relevance:** One respondent skipped the item with the food items.<br>**Change:** A separate sheet with instructions was added. Since there had been a lot of discussions about the number of items and answering options in the table, the research team decided to separate two	**Item 13a**<br>**Does the food item affect your stomach?**
**If the food item affects your/your child's stomach, could you explain in which way?**<br>(Item 12b)		**If yes: In which way**<br>(Item 12b)		**If yes: In which way?**<br>(Item 12b)		**Item 13b**<br>**If yes: In which way?**
**Do you choose specific food items to help your/your child's stomach?**<br>(Item 12c)		**Do you choose specific food items to help your stomach?**<br>(Item 12c)		**Do you choose specific food items to help your stomach?**<br>(Item 12c)		

**Do you avoid specific types of food to help you/your child's stomach?** (Item 12d)	options were decreased in number, as suggested by participants.	**Do you avoid specific types of food to help your stomach?** (Item 12d)		**Do you avoid specific types of food to help your stomach?** (Item 12d)	of the items and place them as individual items.	
**Response options:**	**Comprehension and relevance:** The response options were explained to be easy to understand, but hard to distinguish between two of the options when using the 5 p Likert scale, as one participant stated: "*It's sometimes hard to tell the difference between 'rarely' and 'sometimes'* ". Participants thought that predefined answering options were clear and preferable over a, for example, visual analog scale. The participants also wished to be able to answer the questions using their own phrasing. **Change:** The scale of answering options was changed to a 4 p Likert scale, according to input from participants. The option "*Please explain how*" was added in 10 questions.	**Response options:**	No problems reported.	**Response options:**	No problems reported.	**Response options:**
**Never**		**No, never**		**No, never**		**No, never**
**Rarely**						
**Sometimes**		**Yes, sometimes**		**Yes, sometimes**		**Yes, sometimes**
**Often**		**Yes, often**		**Yes, often**		**Yes, often**
**Always**		**Yes, always**		**Yes, always**		**Yes, always**
**Not currently, but I have in the past**		**Not currently but I have in the past**		**Not currently but I have in the past**		**Not currently but I have in the past**
		**Comments**		**Please explain how**		**Please explain how**
