# Supplementary material for: Diet and bowel function in children with Hirschsprung’s disease: development and content validation of a patient-reported questionnaire
Source: BMC Nutr. 2023 Jun 28;9:78. doi: 10.1186/s40795-023-00737-6 (PMC10308757; doi:10.1186/s40795-023-00737-6)
Supplement: Supplementary file 2 — Additional file 2. [file 40795_2023_737_MOESM2_ESM.pdf]

Additional file 2. The Diet and Bowel Function questionnaire. Final version

Background data	Bowel function	Dietary effects on bowel function and daily life	The effect of specific food items on bowel function
**1. Age**<br>(Years)<br>**2. Gender**<br>(Girl/Boy)<br>**3. Height**<br>(cm)<br>**4. Weight**<br>(kg)<br>**5a. Do you have any disease, allergy or any gastrointestinal congenital malformation?** (Yes/No)<br>**5b. If yes, What allergy/disease?**<br>(Open answer)<br>**5c. What is the gastrointestinal congenital malformation?**<br>(Open answer)<br>**6a. Do you take any treatment for bowel symptoms?**<br>(Yes/No)<br>**6b. If yes, What type of treatment?**<br>(Adjusted diet/Medicine (e.g. for diarrhoea or constipation, antibiotics, analgesic), What type of medicine?/Enemas/Other reason)<br>**6c. If yes, for what reason?**<br>(Constipation/Diarrhoea/Stomach pain/Other reason)<br>**7. Have you ever had impaired growth?** (Weight Yes/no, Height Yes/No)<br>**8. Have you ever had the need for nutritional supplements (e.g. oral nutritional supplements)?**<br>(No, never, Not currently, but I have in the past, Yes, occasionally need, Yes, regularly need)<br>**9a. Do you have any special diet? (e.g. vegetarian, vegan)**<br>(Yes/no)<br>**9b. If yes, What type of special diet?**<br>(Open answer)	**1. Do you experience abdominal pain?** (Never/ Sometimes [at most once a week]/ Frequently/ Always)<br>**2. How often do you/your child poop?** (Every other day to twice a day/More often than twice a day/Less often than every other day)<br>**3. How many times a week do you/your child poop?**<br>(Open answer)<br>**4. Do you/does your child have hard poop and troubles to push it out?** (No/ Yes, but it passes without doing anything/ Yes, then I need to eat prunes, pears, kiwis or other food to make the poop more soft/ Yes, I need to eat medicine that makes the poop softer/ Yes, I need to use medicine in my butt to be able to push the poop out)<br>**5. Do you experience difficulties in passing stools despite soft poop?** (No, never/ Yes, without treatment/ Yes, manageable with diet/ Yes, manageable with medicine/ Yes, not manageable with either diet and/or medicine)<br>**6. Do you suffer from bothersome gases?** (Never/ Sometimes [at most once a week]/ Frequently/ Always)<br>**7. Do you/does your child ever experience difficulties in school or in their spare time, for example doing what you/your child wants, or seeing friends, due to problems with stools coming in underwear or the risk of it happening?** (No social problems/ Sometimes/ Often/ Daily)<br>**8. Do you/does your child feel the urge to poop and verbalizes when you/he or she will poop?**<br>(Always/Most of the time/Not often/seldom/Never)<br>**9. Are you/your child able to hold back defaecation?**<br>(Yes, always or almost always/ Problems less than 1 time per week/ Weekly/ No, my child has no or little voluntary control)<br>**10. Do you/does your child ever soil (staining in underwear)?** (Never or very seldom/ Less than 1 time per week no change of underwear required/ Often, change of underwear required many times per week/Daily, requires protective aids)<br>**11. Do you/does your child ever have accidents where a lot of poop comes in the underwear?**<br>(Never/ Less than 1 time per week/ Weekly, often require protective aids/ Daily, protective aids required day and night)	**1. Would you agree that your diet affects your stomach? (e.g. constipation, diarrhoea or bloatedness)**<br>(No, never/Yes, sometimes/Yes, often/Yes, always/Not currently but I have in the past/Please, explain how)<br>**2. Would you agree that how you eat affects your stomach?**<br>(No, never/Yes, sometimes/Yes, often/Yes, always/Not currently but I have in the past/Please, explain how)<br>**3a. Do you adjust your diet for your stomach's sake?**<br>(No, never/Yes, sometimes/Yes, often/Yes, always/Not currently but I have in the past/Please explain how)<br>**3b. If yes: Why?**<br>(Laxative effect/Constipating effect/Less gases/Other. If so, what?)<br>**4. Do you choose specific types of food to help your stomach?**<br>(Yes/No)<br>**5. Do you avoid specific types of food to help your stomach?**<br>(Yes/No)<br>**6. Is there anyone else in your family who adjusts their diets to help their stomach?**<br>(Yes/No)<br>**7. Does your diet limit you (in school, when you are with friends or in general)?**<br>(I've never thought about it, so I don't find it relevant/No, never/Yes, sometimes/Yes, often/Yes, always/Not currently but I have in the past/Please explain how)<br>**8. Do you think about how your diet affects your stomach?**<br>(No, never/Yes, sometimes/Yes, often/Yes, always/Not currently but I have in the past/Please explain how)<br>**9. To parents: Do you think about your child's diet and how it affects his/her stomach?**<br>(No, never/Yes, sometimes/Yes, often/Yes, always/Not currently but I have in the past/Please explain how)<br>**10. Does your diet affect you emotionally?**<br>(No, never/Yes, sometimes/Yes, often/Yes, always/Not currently but I have in the past/Please explain how)<br>**11. To parents: Does your child's diet affect you emotionally?**<br>(No, never/Yes, sometimes/Yes, often/Yes, always/Not currently but I have in the past/Please explain how)<br>**12a. Would you be interested in finding out more information about how your diet affects your stomach?**<br>(Yes/No)<br>**12b. If yes: Where or who would you turn to to find out more information?**<br>(Open answer)	**13a. Does the food item affect your stomach?**<br>(For every food item: Yes/No/I don't know)<br>**13b. If yes: In which way?**<br>(For every food item: Laxative effect/Constipating effect/Gives gases/Gives pain/Other. If so, what?)<br><br>**Food items listed:**<br>**Fruits**: pineapple, orange, apricot, banana, clementine, strawberry, kiwi, cherry, lingonberry, melon, nectarine, peach, plum, pear, dried fruit, grapes, apple<br>**Vegetables**: avocado, cauliflower, broccoli, beans, cabbage, lentils, onion, corn, carrot, pepper, parsley, potato, rhubarb, celery, fruit peel, asparagus, mushroom, tomato, peas<br>**Dairy**: cream, ice cream, lactose-free milk, milk, cheese, butter, yoghurt<br>**Bread, flour, rice**: bread with grains/seeds, Swedish cracker, cornmeal, pasta, rice, flour, white bread<br>**Meat, fish, egg**: fish, pork, beef, sausage, chicken, salami, shellfish, egg<br>**Sweets and snacks**: pastry, chips, chocolate, candy, popcorn, rice cakes, pretzel sticks<br>**Beverages**: soda (with sugar), soda (free from sugar), water, carbonated drink, wine/beer, formula<br>**Cooking effects**: deep fried food, spicy food, fried food, soup<br>**Spices and seeds**: curry, cayenne, chili/tabasco, chamomilla, sesame, sunflower seeds, poppy seeds<br>**Nuts etc.**: cashew, hazelnut, peanut, chestnut, almond, brazil nut, walnut, soy<br>**Other food item** (Open answer)
